# Acute Post-operative Transient Sialadenitis "Anesthesia Mumps" After Caesarean Section Under Spinal Anesthesia: A Case Report

**DOI:** 10.7759/cureus.44635

**Published:** 2023-09-04

**Authors:** Saba' Jarrar, Shawkat Altamimi, Suzan Damrah

**Affiliations:** 1 Division of Otolaryngology, Head and Neck Surgery, Department of Surgery, Al-Balqa Applied University, Al-Salt, JOR; 2 Obstetrics and Gynaecology, Arab Medical Center, Amman, JOR

**Keywords:** caesarean section, anesthesia spinal, parotid gland swelling, post operative sialadenitis, anesthesia mumps

## Abstract

Acute postoperative transient sialadenitis, also known as anesthesia mumps, is a rare condition that is transient, usually benign, and self-limiting in nature involving unilateral or bilateral parotid glands. The exact mechanism and etiology have not been fully explained, but the causative factors may include, pneumoparotitis, venous congestion, excess saliva secretion, surgical position, perioperative dehydration, and perioperative use of drugs such as atropine, succinylcholine, morphine, ephedrine, and propofol. We report a case of a 31-year-old pregnant lady who was admitted for elective cesarean section under spinal anesthesia. She developed facial swelling involving the parotid area bilaterally five hours postoperatively. Dexamethasone was given intravenously with intravenous (IV) fluids and paracetamol. The swelling improved gradually and resolved completely after 48 hours. This is a case of anesthesia mumps, one of the rare cases that may develop after anesthesia. Awareness, early recognition and supportive management by rehydration and corticosteroids are essential for the patient's reassurance, smooth regression, and recovery, and to avoid extremely rare situations as this condition can progress into airway obstruction.

## Introduction

One of the uncommon side effects of general or even spinal anesthesia is an acute, transient swelling of the salivary glands, particularly the parotid glands, known as "anesthesia mumps," which typically happens during or after surgery [1,2]. The exact incidence is unknown, but it is reported to be 0.16. % to 0.2% [3]. It frequently starts as a painless unilateral or bilateral parotid enlargement that subsides within a few days with no long-term sequelae [4]. Rarely, respiratory distress may arise, necessitating immediate intervention to secure the airway. This is an extremely rare instance, and the exact incidence is unknown, There are two reported cases of airway obstruction with anesthesia mumps, one of which required tracheostomy to secure the airway [5]. Here, we report a case of bilateral parotid swelling post-cesarean section under spinal anesthesia in a young female patient. Anesthesia mumps is usually reported after general anesthesia, and this is one of the few cases that occurred following spinal anesthesia [6]. There is a limited number of publications about anesthesia mumps after spinal anesthesia [2].

## Case presentation

This is a case report of a 31-year-old female patient, Gravid 2 Para 1 Live 1. She didn't have medical illnesses; her previous obstetrical history included a previous C/S delivery of a term newborn under general anesthesia with no post-operative complications; the indication of the previous C/S was failure to progress. Her current pregnancy had been uncomplicated. Her ultrasound studies and routine laboratory tests were normal throughout the pregnancy. She was planned for elective C/S due to previous C/S. Preoperatively, her vital signs were: blood pressure 110/51 mm/Hg, pulse: 81/min, temperature: 36^o^C, respiratory rate: 12/min, O2 saturation 99%. Her blood tests were within the normal range (Table 1).

**Table 1:** Laboratory findings preoperatively and day 1 post-operatively WBC: White Blood Cells Count

	WBC 10^3^/ul	Hemoglobin g/dl	Platelets 10^3^/ul
Preoperatively	8.04	11.7	267
Day 1 post-operatively	9.50	11.6	233

Spinal anesthesia induction was started after taking patient consent at the level of L3-L4 by Marcaine 0.5%. After confirmation of anesthesia, C/S was performed uneventfully. The patient was also given ephedrine 30mg, dexamethasone 8mg, ceftriaxone 1 gm, esomeprazole 40mg, and syntocinon 30IU intravenously. After delivery, the patient was in good condition. Her vital signs at the end of the procedure were: blood pressure (BP) 130/70 mm/Hg, pulse: 88/min, temperature: 36^o^C, respiratory rate: 12/min, and O2 saturation: 100%. Five hours postoperatively, the patient reported to the nurses that she started to have facial swelling involving the parotid areas bilaterally. It was painless, and her vital signs were: BP 120/65, pulse rate 90/min, respiratory rate 19/min, temp 37.2^o^C, O2 saturation 96%. Examination showed bilateral parotid swelling that was more noticeable on the right side, with no overlying redness, hotness, fluctuation, crepitation, or tenderness. The patient was lying on her bed comfortably with no signs of distress, and intraoral examination showed patent bilateral Stensen’s ducts and no purulent discharge. The patient had no previous history of salivary gland diseases. Her blood tests on the first postoperative day were within normal limits (Table 1).

Given the history and clinical findings, the patient was diagnosed with acute bilateral postoperative sialadenitis (anesthesia mumps). IV fluids were given, and she was administered intravenous dexamethasone 8mg every eight hours for 24 hours. Warm compressors were applied. Her condition started to improve the next day, and the swelling subsided in size gradually and resolved completely 48 hours postoperatively.

## Discussion

Anesthesia mumps is an acute postoperative unilateral or bilateral swelling of the parotid gland, first described in 1960 [2,4], and the reported incidence is 0.16. % to 0.2% [3]. The exact pathophysiology of anesthesia mumps is unclear, and many factors are thought to play a role (Table 2).

**Table 2:** Possible Factors that are thought to play a role in the pathophysiology of anesthesia mumps

Possible causative factors	Mechanism
Muscle relaxant usage in general anesthesia	Loss of muscle tone may facilitate the retrograde passage of air into the parotid gland when the positive pressure intraorally is increased during induction of anesthesia or after coughing and excessive straining at the emergence from general anesthesia [3,7].
Perioperative medications like atropine, succinylcholine, glycopyrrolate, and morphine	Retention and increased viscosity of saliva can occlude the salivary ducts [8].
Preoperative dehydration	Retention and increased viscosity of saliva can occlude the salivary ducts [2].
Intravenous ephedrine	Ephedrine has a beta-stimulant effect that can cause vasodilation in the parotid gland [2].
Surgical position	Extreme head rotation in a long-standing surgical procedure can impair the proper drainage of the parotid gland [3] and can potentially cause glandular ischemia by squeezing arterial or venous vessels [5].
Intratracheal manipulation	Stimulation of parasympathetic nerves that mediate the pharyngeal reflex, which induces salivation, vasodilation, and hyperemia in the salivary gland [9].

First, muscle relaxant usage in general anesthesia leads to loss of muscle tone, which may facilitate the retrograde passage of air into the parotid gland when the positive pressure intraorally is increased during induction of anesthesia, in addition to coughing and excessive straining at the emergence from general anesthesia which can cause retrograde airflow into the parotid gland [3,7]. Second, perioperative medications like atropine, succinylcholine, glycopyrrolate, and morphine, in addition to preoperative dehydration, can cause the retention and increased viscosity of saliva, occluding the salivary ducts [8]. The use of intravenous ephedrine which has a beta stimulant effect that can cause vasodilation in the parotid gland [2]. Third, surgical positions like extreme head rotation in a long-standing surgical procedure can impair the proper drainage of the parotid gland [4] or can potentially cause glandular ischemia by squeezing arterial or venous vessels [5]. Fourth, intratracheal manipulation stimulates parasympathetic nerves that mediate the pharyngeal reflex, which induces salivation, vasodilation, and hyperemia in the salivary gland [9].

Prolonged operations under general anesthesia with a prone position or hyperextension of the head contribute to the development of anesthesia mumps. In C/S under spinal anesthesia, the operation is short; the position is supine, so the responsible factors for the development of anesthesia mumps are most likely due to preoperative dehydration and the use of intravenous ephedrine intra-operatively, which has a beta stimulant effect [2]. The number of publications related to anesthesia mumps post-spinal anesthesia is limited. There are few reported cases of anesthesia mumps post neuraxial anesthesia (spinal, epidural, and combined spinal-epidural) [2]. In our case, the past medical history was unremarkable, our patient had no previous salivary gland diseases, and the surgery was short, in the supine position and under spinal anesthesia, so we assume that the use of intravenous ephedrine during anesthesia played the major role in the development of anesthesia mumps in addition to possible preoperative dehydration. Anesthesia mumps is generally a self-limiting disease with a benign course that usually subsides within hours to a few days. The definitive treatment for this condition is still to be found, but administering intravenous fluids, corticosteroids, and anti-inflammatory medications can improve the symptoms [6]. Our patient received intravenous fluids and three doses of 8mg intravenous dexamethasone, and her condition started to improve within less than 24 hours and had completely resolved after 48 hours. Awareness and early recognition of this condition will help to provide supportive treatment. This will help in early airway management in extremely rare cases of massive facial and pharyngeal swelling causing respiratory distress and airway obstruction [5].

## Conclusions

Anesthesia mumps is a rare benign self-limiting condition that can develop after general anesthesia and more rarely after spinal anesthesia. Early recognition and administration of intravenous fluids, corticosteroids, and anti-inflammatory medications can help to improve the symptoms which usually subside within hours to a few days. Surgeons, anesthetists, and postoperative nurses and caregivers should be aware of this condition to reassure the patient and to ensure immediate intervention in extremely rare cases when massive facial and pharyngeal swelling may develop leading to respiratory distress and airway obstruction.
